# Temporal Trends in Multiple Births in Greece: The Evolution of an Epidemic

**DOI:** 10.7759/cureus.35414

**Published:** 2023-02-24

**Authors:** Nikolaos Vlachadis, Dionysios Vrachnis, Nikolaos Loukas, Alexandros Fotiou, Georgios Maroudias, Nikolaos Antonakopoulos, Sofoklis Stavros, Nikolaos Vrachnis

**Affiliations:** 1 Department of Obstetrics and Gynecology, General Hospital of Messinia, Kalamata, GRC; 2 Department of Clinical Therapeutics, National and Kapodistrian University of Athens, Alexandra Hospital, Athens, GRC; 3 Department of Obstetrics and Gynecology, Tzaneio Hospital, Piraeus, GRC; 4 Third Department of Obstetrics and Gynecology, National and Kapodistrian University of Athens, Attiko Hospital, Athens, GRC; 5 Department of Obstetrics and Gynecology, University of Patras, Patras, GRC

**Keywords:** births, greece, assisted reproduction (art), twin births, multiple births

## Abstract

Introduction

Multiple births constitute the dominant adverse effect of fertility treatments and are associated with increased perinatal risks. The aim of this study was to comprehensively examine and present time trends in multiple births in Greece.

Methods

Data on live births by multiplicity were derived from the Hellenic Statistical Authority, covering a 65-year period from 1957 to 2021. Temporal trends in multiple birth rates (MBR), twin birth rates (TwBR), as well as in triplet and higher-order birth rates (Tr+BR) were assessed using joinpoint regression analysis, and the annual percentage changes (APC) were calculated with a 95% confidence interval (95% CI) and level of statistical significance (p < 0.05).

Results

The MBR in Greece showed a downward trend from 1957 to 1979 (APC = -1.7, 95% CI: -2.0 to -1.4, p < 0.001). However, the rate started to climb in the 1980s, accelerated during the 1990s, and continued to rise in the two most recent decades, reaching a historic high and a world record of 57.2 per 1,000 births in 2021, i.e., a 3.4-fold increase since 1985. The TwBR increased from an all-time low of 16.5 per 1,000 births in 1978 with APC = 1.4 (95% CI: 0.2 to 2.5, p = 0.021) during 1979-1989, APC = 6.3 (95% CI: 5.5 to 7.2, p < 0.001) during 1989-2001, and APC = 1.2 (95% CI: 0.8 to 1.5, p < 0.001) during the last two decades (2001-2021). The Tr+BR, after an all-time low of 17.5 per 100,000 births in 1966, increased dramatically from 1982 to 2000 (APC = 12.4, 95% CI: 9.6 to 15.2, p < 0.001), leveled off during 2000-2011, and after reaching a historic maximum of 351.1 per 100,000 births in 2010, there was a sharp decreasing trend during the last decade (2011-2021: APC = -12.1, 95% CI: -16.8 to -7.2, p < 0.001).

Conclusion

The dramatic increases in maternal age as well as in medically assisted conceptions have resulted in an epidemic increase in MBR in Greece reaching world record levels. During the last decade, there was an encouraging decline in the Tr+BR; however, the TwBR has continued to trend upwards.

## Introduction

Multiple pregnancies, most commonly twins and less commonly triplets or higher-order, result either from multiple ovulation and fertilization of more than one egg (dizygotic twins) or from the formation of two embryos after the spontaneous separation of a single embryo in the early stages of development (monozygotic or identical twins). The incidence of multiple births after natural conception is generally less than 2%, with the exception of certain population groups in Africa [1,2]. Apart from racial and genetic factors, as well as the woman’s somatometry and family history, the main epidemiologic factor increasing the frequency of multiple births is the advancing maternal age. The incidence of monozygotic twins is considered to be globally stable at about four per 1,000 births, thus the variation in the incidence of multiple births is apparently the result of a corresponding change in the incidence of dizygotic pregnancies [2-4].

The incidence of multiple births has generally declined since the 1950s reaching a minimum in the late 1970s, probably due to the progressive decline in maternal age, but has been steadily increasing since then. This rise occurred largely due to the social trend toward delayed childbearing and advanced maternal age; however, the main factor in this increasing trend was iatrogenic. In the last four decades, the world has witnessed a rapid development of assisted reproduction technology (ART) with treatments that have effectively helped numerous couples with infertility. Fertility treatments mainly include in vitro fertilization (IVF) and intracytoplasmic sperm injection (ICSI), as well as non-IVF/ICSI procedures, which include drugs administration for ovulation induction or ovarian stimulation for producing multiple ovulation, in combination with timed intercourse or intrauterine insemination. However, the practice of transferring more than one embryo, as well as drug induction of multiple ovulation, has led to a huge increase in multiple births in many developed countries [2-5]. Although IVF/ICSI is associated with a more than doubling of monozygotic twins, the vast majority of these extra twin births are dizygotic [6].

Multifetal gestations have a significantly increased risk of poor perinatal outcomes and involve substantial healthcare costs [7]. Specifically, they are strongly associated with increased incidence of all major obstetric complications, including fetal congenital anomalies, fetal death, premature delivery, fetal growth restriction, preeclampsia, and gestational diabetes [8].

Greece is a country characterized by advanced maternal age, low birth rate, and increased use of medically assisted reproduction [9-11] with an extremely high preterm birth rate [12,13]. Thus, we aimed to comprehensively investigate the temporal trends of multiple births in the Greek population.

## Materials and methods

Official national data regarding live births in Greece by multiplicity, based on the birth certificates registered in the country, were retrieved from the Hellenic Statistical Authority, covering a 65-year period from 1957 to 2021, the first and the most recent years with fully available data, respectively.

For each year of the above period, the multiple birth rate (MBR), defined as the number of live births in multiple deliveries per 1,000 live births, the twin birth rate (TwBR), defined as the number of live births delivered from twin pregnancies per 1,000 live births, as well as the triplet and higher-order birth rate (Tr+BR), defined as the number of live births from triplet and other higher-order multiple deliveries per 100,000 live births, were calculated. The unit of analysis for live birth was the live-born neonate, not the delivery, according to the definitions suggested by the United States Centers for Disease Control and Prevention [14].

Trends in the rates of multiple births (MBR, TwBR, and Tr+BR) were assessed using Joinpoint regression software version 4.7.0.0 (Surveillance Research Program, National Cancer Institute, Bethesda, MD). The annual percentage changes (APCs) were calculated with a 95% confidence interval (95% CI) and level of statistical significance (p < 0.05).

Since this was an analysis of national-aggregate publicly available data, ethics board approval or consent procedures were not needed.

## Results

During the period from 1957 to 2021, a total of 7,910,174 live births were registered in Greece, of which, 7,676,489 were single births and 233,685 were multiple births, resulting in a total MBR of 29.5 per 1,000 births. Among multiple births, the majority (225,949 or 96.7%) were twins, 7,500 were triplets (3.2%), and 236 (0.1%) were quadruplets and higher-order multiple births. The overall TwBR was 28.6 per 1,000 live births, and the overall T+BR was 97.8 per 100,000 births.

**Figure 1:**
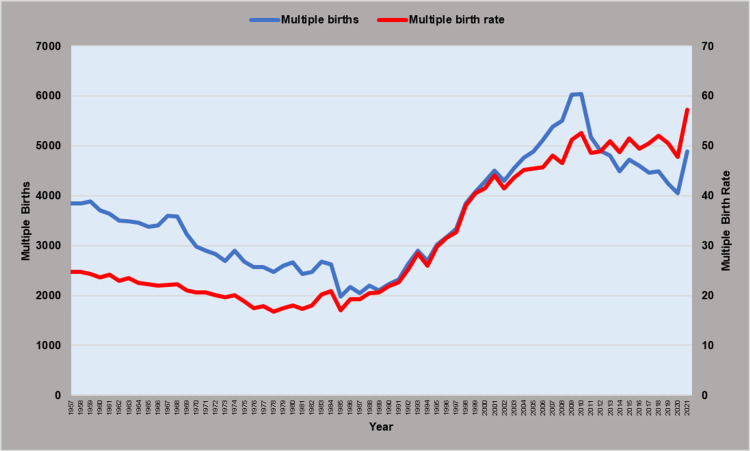
Multiple births and multiple birth rates (per 1,000 births) in Greece, 1957-2021

**Figure 2:**
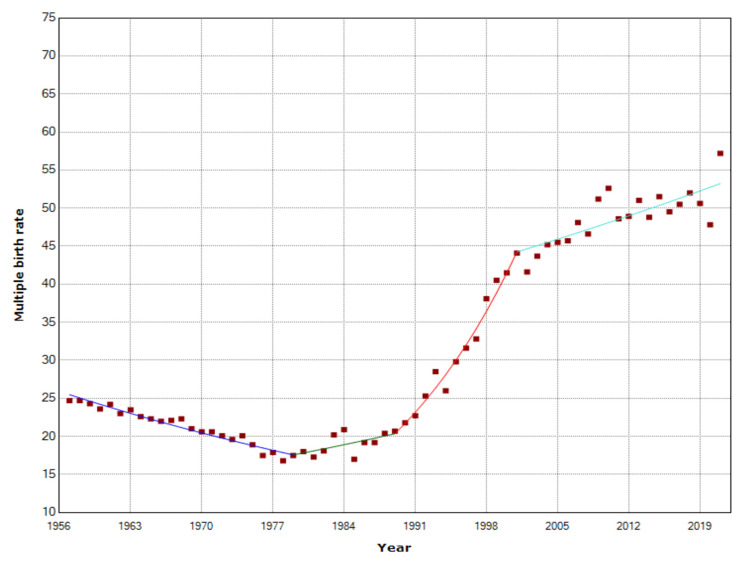
Temporal trends in multiple birth rates (per 1,000 births) in Greece, 1957-2021

The MBR showed a downward trend from 1957 to 1979 (APC = -1.7, 95% CI: -2.0 to -1.4, p < 0.001). However, during the 1980s, the trend began to rise (1979-1989: APC = 1.5; 95% CI: 0.3 to 2.7, p = 0.014), and in the following decade of the 1990s, the MBR showed a steep upward trend (1989-2001: APC = 6.7, 95% CI: 5.8 to 7.6, p < 0.001), which continued at a slower pace in the last two decades (2001-2021: APC = 0.9, 95% CI: 0.6 to 1.3, p < 0.001). The MBR fell 32%, from 24.7 per 1,000 births in 1957 to the historic minimum of 16.8 per 1,000 births in 1978, and to 17.0 per 1,000 births in 1985, while after a 3.4-fold increase, it reached the historic maximum of 57.2 per 1,000 births in 2021. In 1985, it was an all-time low of multiple births (1,986), whereas, in 2010, it was the all-time zenith with 6,042 multiple births. In the last decade, although the MBR continued the rising trend, the number of multiple births largely decreased, reaching 4,048 in 2020, 33% down from 2010 (Figures 1, 2 and Table 1).

**Table 1:** Temporal trends in multiple birth rates in Greece, 1957-2019 APC: annual percentage change.

Period	APC	95% CI	P-value
1957-1979	-1.7	-2.0 to -1.4	<0.001
1979-1989	1.5	0.3 to 2.7	0.014
1989-2001	6.7	5.8 to 7.6	<0.001
2001-2021	0.9	0.6 to 1.3	<0.001

**Figure 3:**
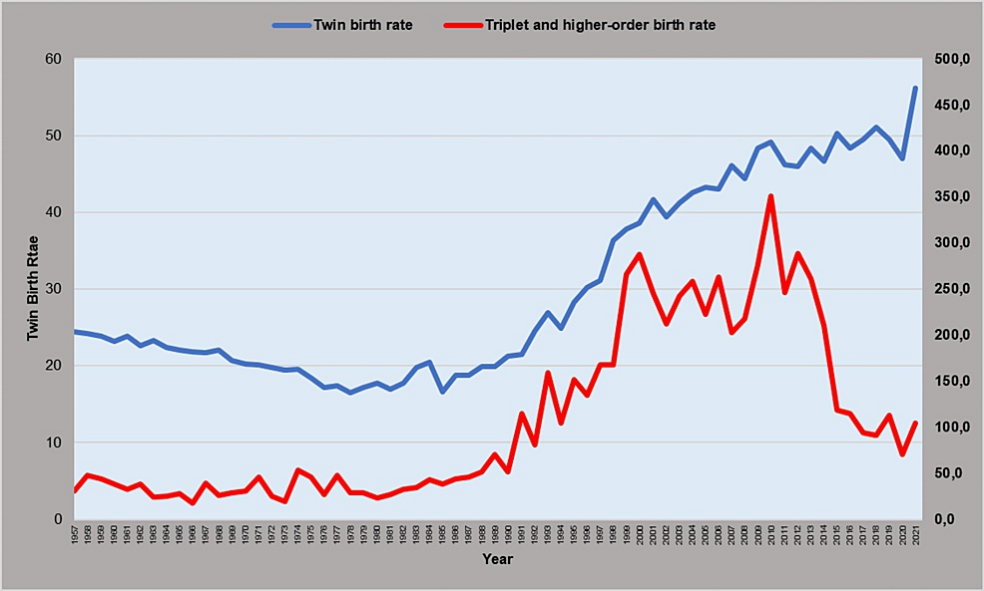
Twin birth rates (per 1,000) and triplet and higher-order birth rates (per 100,000) in Greece, 1957-2021

**Figure 4:**
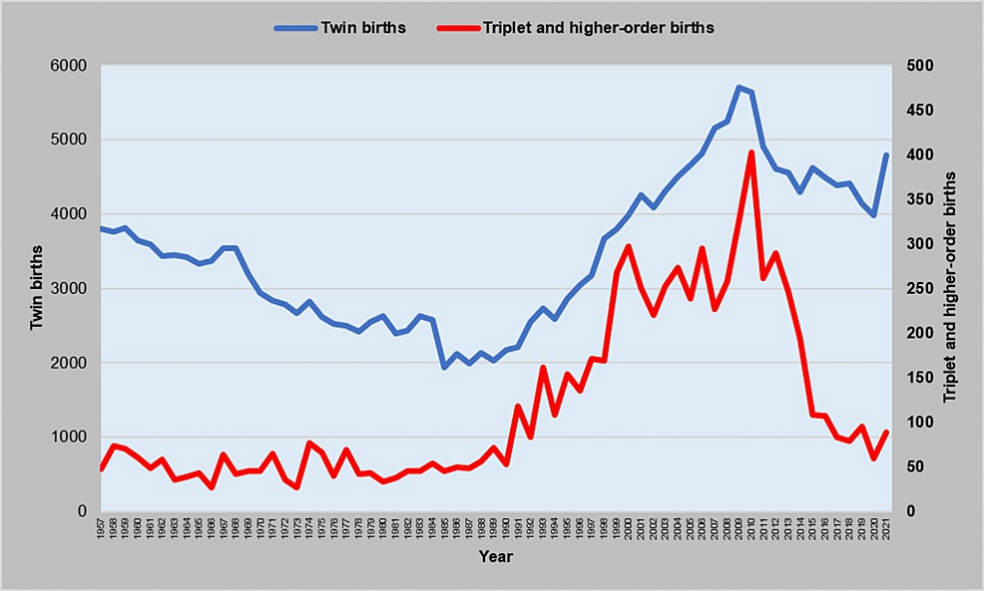
Twin births and triplet and higher-order births in Greece, 1957-2021

**Figure 5:**
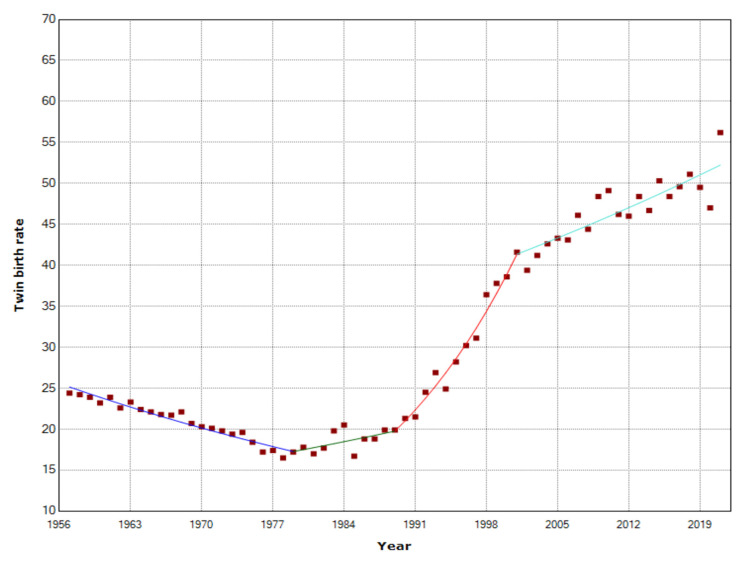
Temporal trends in twin births rates (per 1,000 births) in Greece, 1957-2021

The trend for the TwBR was downward with APC = -1.7 (95% CI: -2.0 to -1.4, p < 0.001) in the period 1957-1979, with a historic low of 16.5 per 1,000 births in 1978, and then upward, with APC = 1.4 (95% CI: 0.2 to 2.5, p = 0.021) during 1979-1989, APC = 6.3 (95% CI: 5.5 to 7.2, p < 0.001) during 1989-2001, and APC = 1.2 (95% CI: 0.8 to 1.5, p < 0.001) in the last two decades (2001-2021), reaching a historic high of 56.2 twin births per 1,000 births in 2021 (Figures 3, 4 and Table 2). The historically lowest number of twin births in Greece was 1,941 in 1985, whereas the record high was in 2009 with 5,709 twin births, followed by a 30% reduction to 3,988 in 2020 (Figures 3-5 and Table 2).

**Table 2:** Temporal trends in twin birth rates in Greece, 1957-2019 APC: annual percentage change.

Period	APC	Lower CI	P-value
1957-1979	-1.7	-2.0 to -1.4	<0.001
1979-1989	1.4	0.2 to 2.5	0.021
1989-2001	6.3	5.5 to 7.2	<0.001
2001-2021	1.2	0.8 to 1.5	<0.001

**Figure 6:**
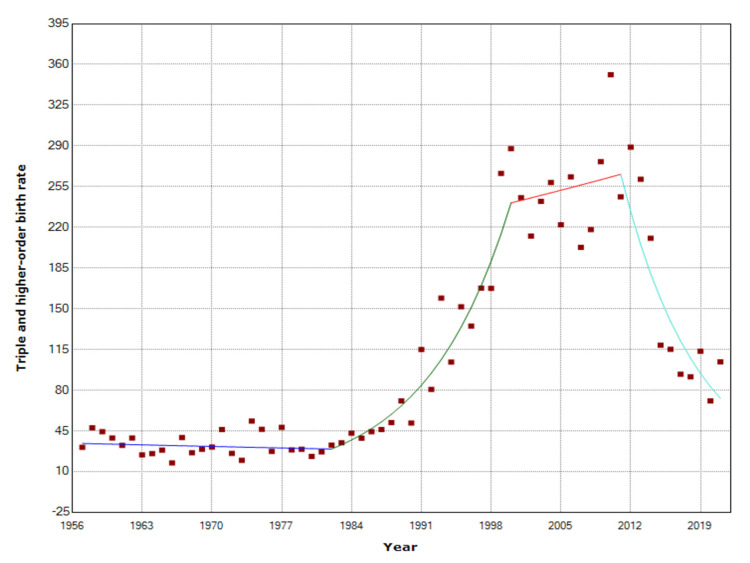
Temporal trends in triple and higher-order birth rates (per 100,000 births) in Greece, 1957-2021

The Tr+BR remained unchanged during 1957-1982, with an all-time low of 17.5 per 100,000 births in 1966, but a steep increasing trend until 2000 followed, with APC = 12.4 (95% CI: 9.6 to 15.2, p < 0.001). The rate leveled off during 2000-2011, and this was followed by a sharp decreasing trend during the last decade (2011-2021: APC = -12.1, 95% CI: -16.8 to -7.2, p < 0.001). In 2010, the Tr+BR reached a historic maximum of 351.1 per 100,000 births, a 20-fold increase compared with 1966, and a nine-fold up from 1985, whereas it fell five-fold to 70.8 per 100,000 births in 2020. The number of triplet and higher-order births ranged substantially from 27 in 1973 (minimum) to the all-time maximum of 403 in 2010, i.e., a 15-fold increase and nine times higher than in 1985 (Figures 3, 4, 6 and Table 3).

**Table 3:** Temporal trends in triplet and higher-order births in Greece, 1957-2019 APC: annual percentage change.

Period	APC	Lower CI	P-value
1957-1982	-0.6	-2.0 to 0.8	0.389
1982-2000	12.4	9.6 to 15.2	<0.001
2000-2011	0.9	-4.5 to 6.6	0.750
2011-2021	-12.1	-16.8 to -7.2	<0.001

In 2021, there were sharp increases of 16% in MBR (from 47.8 to 57.2 per 1,000 births) and TwBR (from 47.0 to 56.2 per 1,000 births), and 32% in Tr+BR (from 70.8 to 104.3 per 100.000 births), compared with 2020. From 2020 to 2021, the multiple and twin births rose 17% (from 4,048 to 4,884, and from 3,988 to 4,795, respectively), and the triplet and higher-order births escalated by 48% (from 60 to 89), compared with a slight 0.3% decline in single births (from 80,716 to 80,462).

## Discussion

In the present study, the time trends of multiple birth rates in Greece over a period of 65 years were thoroughly examined from 1957 (the year with the oldest available complete data) to 2021 (the most recent year with finally available data). Our analysis revealed hefty increasing trends of multiple births in the Greek population, which have reached epidemic levels. The increase started in the 1980s, accelerated during the 1990s, and continued steadily, albeit at a slower pace, in the two most recent decades, with the MBR reaching its historic high in 2021. Trends in TwBR have generally paralleled those of MBR, given that twin births account for the vast majority of multiple births. In contrast, the Tr+BR, after a steep upward trend in the 1980s and 1990s, remained essentially unchanged in the 2010s and recorded a notable downward trend in the most recent decade.

The longitudinal trends in the rates of multiple births can be divided into two distinct periods: before and after the implementation of medically assisted reproduction methods. Fertility treatments are the main reason for the increase in multiple births worldwide. In IVF/ICSI, multiple births primarily result from the transfer of more than one embryo during the procedure, whereas non-IVF/ICSI infertility therapies also confer a substantial proportion of multiple births due to the frequent induction of multiple ovulation [3,8]. It has been estimated that by 2011 in the United States, 36% of twin births and 77% of triplet and higher-order births were attributable to ART procedures, with the non-IVF/ICSI treatments of ovulation induction and ovarian stimulation being the major contributor to the medically assisted multiple births, especially the higher-order multiple births [3]. Moreover, the proportion of multiple births attributable to medically assisted conception increased with maternal age [15].

In Greece, the MBR trend was steadily declining until 1980, by 1.7% annually. This fall was also reported in many other countries [2] and can be explained by the corresponding decline in the maternal age in the Greek population, which reached a historic low in 1980 [11,16]. Of note, this declining trend was only for twin births, whereas the Tr+Br remained unchanged. In the 1970s, the MBR ranged around 20 per 1000 births; this figure represents the frequency of multiple births by natural conception in the Greek population. The MBR reached a historic low of 16.8 per 1,000 births in 1978, accompanied by a historic TwBR low of 16.5 per 1,000 births, whereas the lowest Tr+BR was observed in 1966, with 17.5 per 100,000 births. These numbers were last recovered in 1985, while since then the upward trend has been irreversible, appearing most sharply in the 1990s, with an annual increase of 6.7%. During the first two decades of the 21st century, the increasing trend of MBR has continued steadily, albeit at a lower pace, until today, reaching an unprecedented record level of 57.2 multiple births per 1,000 in 2021.

From 2000 to the present, the longitudinal trends of the rates of twins and triplets and higher-order multiple births have followed distinct patterns. In particular, the TwBR has continued to rise with an APC of 1.2%, reaching an astonishing level of 56.2 per 1,000 births, increased by a factor of 3.4, compared with 1985, while the Tr+BR leveled off during 2001-2011, with an all-time record of 351.1 per 100,000 births in 2010, and then substantially decreased during the last decade by 12.1% annually. In 2020, the Tr+BR in the country was the lowest since 1989, five times less than in 2010, as a result of the delayed implementation of the new guidelines regarding the number of embryos transferred after IVF/ICSI that first emerged in the United States in 1998 [4,8,17]. Besides, the number of multiple births has dropped by one-third during the last decade.

From 2020 to 2021, there was a tremendous rise in the TwBR and the Tr+BR by 16% and 32%, respectively, probably as a result of a recovery of fertility treatments in the country due to the negative impact of the SARS-CoV-2/COVID-19 pandemic on ART activity [18,19]. In 2021, the MBR in Greece reached an all-time record; approximately one in 17 neonates in the country was born from a multiple-gestation pregnancy, presumably the world's highest rate at a country level, surpassing even the African countries [20].

In the last two decades in Europe, there has been a large decrease in transfers of three or more embryos, with a parallel increase in single embryo transfers, especially for younger women with good prognoses. However, Greece has the highest proportion of women aged 40 years or older undergoing IVF/ICSI treatments among the European countries, and a percentage of transfers of three or more embryos that is almost three-fold higher than the European mean [21]. For comparison, in the United States, the 2021 TwBR was 31.2 per 1,000 births, the lowest in almost two decades, whereas the Tr+Br was 80.0 per 100,000 births [14].

Twin pregnancies are associated with a substantially higher risk of poor perinatal outcome than singletons, with this risk rising further as the order of multiples rises. In the United States, compared with singletons, the 2021 preterm birth rate (birth before 37 gestational weeks) was 7 and 11.2 times greater among twins and triplets, respectively, and the 2021 low birth weight (less than 2,500 grams) was 8.1 and 13.8 times higher for twins and triplets, respectively [14]. Furthermore, the 2020 fetal mortality rates for twins and for triplet or higher-order births were 2.2 and 5.2 times higher than that for singletons, respectively [22]. In a study of 2010 data from 29 European countries (not including Greece), compared with singletons, multiple births had a nine-fold increased risk of preterm birth, whereas the risk for neonatal death and fetal death was increased by a factor of 7 and 2.4, respectively [23]. In Greece, an epidemic of preterm births, the leading determinant of neonatal mortality [24], has been developing in parallel with that of multiple births [12]. In 2008, the proportion of the overall preterm birth rate in the country attributable to multiple births was 25.7% [25].

Despite the general consensus that twin pregnancies after IVF/ICSI constitute an adverse outcome to be avoided, it has also been suggested that twin births may represent a favorable and cost-effective outcome of infertility therapies for couples who desire more than one child since the IVF/ICSI twins are associated with 40% lower risks than those conceived naturally [26]. Twin births can play a role in increasing births in Greece where fertility rates are desperately low and there is a high use of ART. The recent increase in births in the country from a historic low in 2019 is attributed entirely to the increase in multiple births.

After 2008, the financial crisis brought negative consequences on the health of the Greek population, especially in fertility rates [27,28]; however, as shown by our results, the MBR remained unaffected. A study of national data for the time period from 1996 to 2015 reported that higher maternal education and socioeconomic status are associated with higher twin rates in Greece [29], i.e., the social groups least affected by economic austerity.

This report is limited to the presentation of the time trends in multiple birth rates in the Greek population. Further study is needed to evaluate the impact of the rising multiple birth rates in Greece on the major complications of pregnancy, including preterm births, low birth weight, fetal deaths, and perinatal morbidity and mortality. The decline in the rates of triplet and higher-order multiple births over the past decade, which are associated with the worst perinatal outcomes, is encouraging. Furthermore, the absolute number of multiple births in Greece decreased during the last decade in parallel with the descending trend in total births. However, the continuing upward trend in the twin birth rate remains alarming and has become a major public health issue for the country.

## Conclusions

The dramatic increases in maternal age as well as in medically assisted conceptions have resulted in an epidemic increase in multiple birth rates in Greece reaching world record levels. Sustained efforts in comprehensive ART surveillance, limiting the number of embryos transferred, increased awareness of multiple births resulting from non-IVF fertility treatments, as well as adequate public information on the possible risks of delayed childbearing are required to curb the multiple birth epidemic in Greece.
